# Adipocyte deficiency of ACE2 increases systolic blood pressures of obese female C57BL/6 mice

**DOI:** 10.1186/s13293-019-0260-8

**Published:** 2019-09-04

**Authors:** Robin Shoemaker, Lisa R. Tannock, Wen Su, Ming Gong, Susan B. Gurley, Sean E. Thatcher, Frederique Yiannikouris, Charles M. Ensor, Lisa A. Cassis

**Affiliations:** 1 Department of Dietetics and Human Nutrition, University of Kentucky, Lexington, KY 40506 USA; 2 Division of Endocrinology and Molecular Medicine, University of Kentucky, Lexington, KY 40536 USA; 3 Department of Physiology, University of Kentucky, Lexington, KY 40536 USA; 4 Division of Nephrology and Hypertension, Oregon Health and Science University, Portland, OR 97239 USA; 5 Department of Pharmacology and Nutritional Sciences, University of Kentucky, Lexington, KY 40536 USA

**Keywords:** Angiotensin-converting enzyme 2, Angiotensin-(1-7), Transgender, Obesity, Blood pressure

## Abstract

**Background:**

Obesity increases the risk for hypertension in both sexes, but the prevalence of hypertension is lower in females than in males until menopause, despite a higher prevalence of obesity in females. We previously demonstrated that angiotensin-converting enzyme 2 (ACE2), which cleaves the vasoconstrictor, angiotensin II (AngII), to generate the vasodilator, angiotensin-(1-7) (Ang-(1-7)), contributes to sex differences in obesity-hypertension. ACE2 expression in adipose tissue was influenced by obesity in a sex-specific manner, with elevated ACE2 expression in obese female mice. Moreover, estrogen stimulated adipose ACE2 expression and reduced obesity-hypertension in females. In this study, we hypothesized that deficiency of adipocyte ACE2 contributes to obesity-hypertension of females.

**Methods:**

We generated a mouse model of adipocyte ACE2 deficiency. Male and female mice with adipocyte ACE2 deficiency or littermate controls were fed a low (LF) or a high fat (HF) diet for 16 weeks and blood pressure was quantified by radiotelemetry. HF-fed mice of each sex and genotype were challenged by an acute AngII injection, and blood pressure response was quantified. To translate these findings to humans, we performed a proof-of-principle study in obese transwomen in which systemic angiotensin peptides and blood pressure were quantified prior to and after 12 weeks of gender-affirming 17β-estradiol hormone therapy.

**Results:**

Adipocyte ACE2 deficiency had no effect on the development of obesity in either sex. HF feeding increased systolic blood pressures (SBP) of wild-type male and female mice compared to LF-fed controls. Adipocyte ACE2 deficiency augmented obesity-induced elevations in SBP in females, but not in males. Obese female, but not obese male mice with adipocyte ACE2 deficiency, had an augmented SBP response to acute AngII challenge. In humans, plasma 17β-estradiol concentrations increased in obese transwomen administered 17β-estradiol and correlated positively with plasma Ang-(1-7)/AngII balance, and negatively to SBP after 12 weeks of 17β-estradiol administration.

**Conclusions:**

Adipocyte ACE2 protects female mice from obesity-hypertension, and reduces the blood pressure response to systemic AngII. In obese transwomen undergoing gender-affirming hormone therapy, 17β-estradiol administration may regulate blood pressure via the Ang-(1-7)/AngII balance.

**Electronic supplementary material:**

The online version of this article (10.1186/s13293-019-0260-8) contains supplementary material, which is available to authorized users.

## Background

Obesity is a primary contributor to the development of hypertension in men and women [1, 2]. Although women have increased adiposity compared to men [3, 4], the prevalence of hypertension is greater in men *versus* women until menopause [5]. After menopause, the prevalence of obesity and hypertension increases in women [5], suggesting sex hormone-mediated mechanisms contribute to protection from obesity-associated hypertension in women.

The renin-angiotensin system (RAS) plays a primary role in regulating blood pressure. Activation of the RAS with obesity contributes to hypertension in experimental models [6, 7] and in humans [8, 9]. Adipose tissue expresses components of the RAS necessary for the production of the vasoconstrictor peptide, angiotensin II (AngII) [10]. Studies from our laboratory demonstrated that adipose tissue serves as a primary source of elevated plasma concentrations of AngII in obese male mice with hypertension [7]. However, this finding may be specific to males, as other studies demonstrated that obese female mice with lower blood pressure than males displayed no increase in plasma AngII concentrations compared to low fat (LF)-fed controls [11]. Rather, obesity in female mice was associated with increased plasma concentrations of the vasodilator peptide, angiotensin-(1-7) (Ang-(1-7)) [11]. Further, compared to lean females, obese female mice had increased adipose tissue expression of angiotensin-converting enzyme 2 (ACE2), a monocarboxypeptidase that cleaves AngII to generate Ang-(1-7) [11]. Whole-body deficiency of ACE2 converted obese female mice to a hypertensive phenotype, elevating blood pressure to the level of obese males [11]. These results suggest that the balance of Ang-(1-7) to AngII, regulated by ACE2, is different in males versus females, contributing to sex differences in the development of obesity-hypertension.

To define mechanisms for sex differences in obesity-hypertension, studies examined the effects of estrogens to regulate the balance of systemic and/or local concentrations of Ang-(1-7) to AngII. 17-β estradiol increased ACE2 mRNA abundance in 3T3-L1 adipocytes (a mouse embryonic fibroblast cell line that can be induced to differentiate into adipocyte-like cells) through an estrogen receptor alpha (ERα)-mediated mechanism [11]. Moreover, administration of 17-β estradiol to ovariectomized obese female mice increased adipose ACE2 mRNA abundance, lowered plasma AngII concentrations and decreased systolic blood pressure [12]. However, 17-β estradiol administration had no effect on these parameters in ovariectomized obese females that were ACE2 deficient, suggesting that protective effects of 17-β estradiol to prevent obesity-hypertension in females were ACE2-mediated. Taken together, these data suggest that estradiol stimulates ACE2 expression in adipocytes to increase the balance of Ang-(1-7) to AngII and protect females from obesity-hypertension.

In this study, we hypothesized that ACE2 expression in adipocytes protects female mice from hypertension associated with obesity. To test this hypothesis, we developed a murine model of adipocyte ACE2 deficiency and used this model to examine effects of adipocyte ACE2 deficiency on the development of hypertension in female and male mice made obese by consumption of a high-fat (HF) diet. Further, to relate these findings to humans, we performed a proof-of-principle study examining the associations among blood pressure, systemic concentrations of estradiol, and the Ang-(1-7)/AngII balance in a patient population of transgendered women (biological males) receiving estradiol therapy.

## Methods

### Experimental animals

All studies using mice were approved by an Institutional Animal Care and Use Committee at the University of Kentucky and were conducted in accordance with the National Institutes of Health (NIH) Guide for the Care and Use of Laboratory Animals. Female mice with loxP sites flanking exon 4 of the *Ace2* gene on a C57BL/6 background (*Ace2*^*fl/fl*^) were bred to male *Ace2*^*fl/y*^ hemizygous transgenic mice expressing Cre recombinase under the control of the adipocyte-specific promoter, adiponectin. The resulting offspring were either experimental animals with adipocyte-ACE2 deletion (*Ace2*^Adipo^) or littermate controls (females *Ace2*^*fl/fl*^; males *Ace2*^*fl/y*^). Mice were maintained on a standard murine diet (Harlan Laboratories, Indianapolis, IN) until 8 weeks of age.

Initial studies characterized the efficiency and specificity of adipocyte ACE2 deficiency using 8-week-old male and female mice (*n* = 7–8 mice per genotype). Kidney, heart, liver, subcutaneous (SubQ), and retroperitoneal fat (RPF) were dissected, frozen in liquid nitrogen, and stored at − 80 °C until use. For Cre expression studies, female mice carrying the transgene with the ROSA26-stop-lacZ reporter (Jackson Laboratory, Bar Harbor, ME, stock # 0003474) were bred to male *Ace2*^Adipo^ mice.

For blood pressure studies, 8-week-old male and female mice of each genotype were randomly assigned to receive ad libitum either a low fat (LF, 10% kcal from fat; D12450B, Research Diets Inc, New Brunswick, NJ) or a high fat diet (HF, 60% kcal from fat; D12492, Research Diets, New Brunswick, NJ) for 4 months (*n* = 6–13 mice/genotype/diet group). Bodyweight was quantified weekly. Fat and lean mass were measured at week 14 of diet feeding by EchoMRI (EchoMRI-100TM, Echo Medical Systems, Houston, TX). Blood pressure was measured by radiotelemetry in a subset of mice (*n* = 5 mice per genotype/diet group) at week 16 of diet feeding for 5 consecutive days, and again following acute administration of AngII (subcutaneous, 20 μg/kg). The method for blood pressure measurement is described previously [13]. Briefly, anesthetized (isoflurane, to effect) mice were implanted with carotid artery catheters advanced to the aortic arch and radiotelemeter implants (model PA-C10) inserted in a subcutaneous pocket on the right flank. After 1 week of recovery, blood pressure was monitored continuously, with values reported every 5 s. Inclusion criteria for blood pressure measurements were (1) pulse pressures > 20 mmHg and (2) pulse pressures > 1 standard deviation of the mean. At study endpoint, mice were anesthetized with ketamine/xylazine (100/10 mg/kg, i.p.) for exsanguination and tissue harvest.

### Acute administration of AngII

HF-fed female *Ace2*^*fl/fl*^ and *Ace2*^Adipo^ and male *Ace2*^*fl/y*^ and *Ace2*^Adipo^ mice (*n* = 4 mice per group) with radiotelemetry implants were subcutaneously (interscapular) administered 20 μg/kg of AngII (Sigma-Aldrich) in 0.9% sterile saline. Blood pressure was recorded via telemetry continuously for 60 min after administration of AngII. Baseline (time = 0 min) blood pressure reported is the average blood pressure over 15 min prior to administration of AngII. Blood pressure at time = 2, 5, 10, 15, 20, 30, 40, 50, and 60 min following AngII administration is the average value per minute. Data are reported as time course and as integrated area under the curve (AUC).

### Detection of β-galactosidase activity in tissues

Whole organs were fixed in formalin at 4 °C for 1 h, then rinsed three times with buffer (100 mM sodium phosphate, 2 mM MgCl_2_, 0.01% sodium deoxycholate, 0.02% NP-40). Organs were incubated overnight in X-gal staining buffer (rinse buffer with 5 mM potassium ferricyanide, 5 mM potassium ferrocyanide, 1 mg/mL X-gal) and then visualized, where blue staining indicates expression of Cre recombinase.

### Tissue DNA and RNA extraction and PCR

Adipose tissue genotyping was performed using DNA extracted from RPF (DNeasy, Qiagen, Alameda, CA). cDNA was generated using the forward primer: 5′–AGCTCATAGAGAAAGAGGGAGCACG and either the reverse primer: 5′–ACAGCCAGGGTGATACAGAGAAACC (generates products demonstrating the presence [912 bp] or the absence [723 bp] of the floxed ACE2 gene) or the reverse primer 5′–AAGGGTAATGTGTGAGCTGGAACCC (generates a 912 bp product demonstrating the deletion of exon 4 of the ACE2 gene).

Total RNA was extracted from tissues using the Maxwell RSC (Promega, Madison, WI). RNA concentrations were determined using a NanoDrop 2000 spectrophotometer (Thermo Scientific, Wilmington, DE); 400 ng of RNA was used for reverse transcription to make cDNA using qScript cDNA Supermix (Quanta, Gaithersburg, MD). The following mouse primers were used to probe gene products from cDNA amplified using SYBR Green PCR Master Mix (Quanta, Gaithersburg, MD): ACE2, forward 5′–TCCAGACTCCGATCATCAAGC, reverse 5′–GCTCATGGTGTTCAGAATTGTGT; 18S, forward 5′–CGGCTACCACATCCAAGGAA, reverse 5′–GCTGGAATTACCGCGGCT. Data are expressed as ΔΔCt relative to 18S rRNA.

### Studies in humans

This study was approved and work was completed in compliance with approval from the Institutional Review Board of the University of Kentucky. Study participants were transgender women (biological males) seeking gender-affirming hormone therapy recruited from the endocrine clinic at the University of Kentucky (*n* = 4 subjects). Inclusion criteria were biologic male subjects between the age of 21 and 60 with a body mass index (BMI) between 30 and 45 kg/m^2^ seeking initiation of estrogen therapy for the first time. Exclusion criteria were fasting blood sugar > 126 mg/dL, or use of diabetes medications, current use of angiotensin-converting enzyme (ACE) inhibitors or angiotensin I receptor blockers (ARBs), anti-inflammatory medications (e.g., steroids), prior estrogens, or any other medication or condition that may affect the RAS pathway. Note that subjects participating in this study delayed the use of spironolactone until after at least 12 weeks of estradiol therapy. Subjects were in overall good health and had no significant hepatic, cardiac, or renal impairment. Subjects were seen at baseline (prior to initiation of estrogen therapy) and 12 weeks after estradiol treatment (estradiol, 1–2 mg/day, orally, dose determined by the endocrinologist). Blood pressure and anthropometric measurements took place during office visits at the endocrine clinic. Blood pressure was measured by arm cuff in the seated and resting position. Blood collection took place at the outpatient Clinical Service Core (CSC) of the institutional Center for Clinical and Translational Science (CCTS). For blood collection, subjects were fasted overnight and arrived at the outpatient CSC at 8am. Plasma was collected after centrifugation and stored at − 80 °C until analysis.

### Quantification of plasma parameters in humans

Estradiol concentrations were quantified using a commercial ELISA kit (Calbiotech, ES180S, Spring Valley, CA; analytical sensitivity of 3 pg/mL). Angiotensinogen concentrations were quantified using a commercial kit (IBL, 27412, Minneapolis, MN; analytical sensitivity of 0.03 ng/mL). Ang-(1-7) peptide concentrations were quantified using a commercial kit (Peninsula Labs, San Carlos, CA, S-1330; analytical sensitivity of 0.01 ng/mL). Plasma renin activity (IBL, IB59131; analytical sensitivity of 0.14 ng/mL) and AngII peptide concentrations were quantified by enzyme- and radioimmunoassay, respectively, as described previously [6, 13, 14].

### Statistical analyses

Data are presented as mean ± SEM. Statistical analyses were performed using SigmaPlot version 12.3. All data passed normality or equal variance tests or logarithmic transformation was used to achieve normality. Two-tailed Student’s *t* tests were used for the analysis of data between two groups. For two-factor analysis, a two-way ANOVA was used to analyze end-point measurements with between-group factors of genotype and diet, followed by Holm-Sidak for post hoc analyses. Response to acute AngII administration was analyzed as a time course using repeated measures (RM) two-way ANOVA, and as the integrated area under the curve (AUC). Correlation analyses were performed for plasma parameters and blood pressures of humans. Values of *p* < 0.05 were considered to be statistically significant.

## Results

### Development of a mouse model of adipocyte ACE2 deficiency

**Fig. 1:**
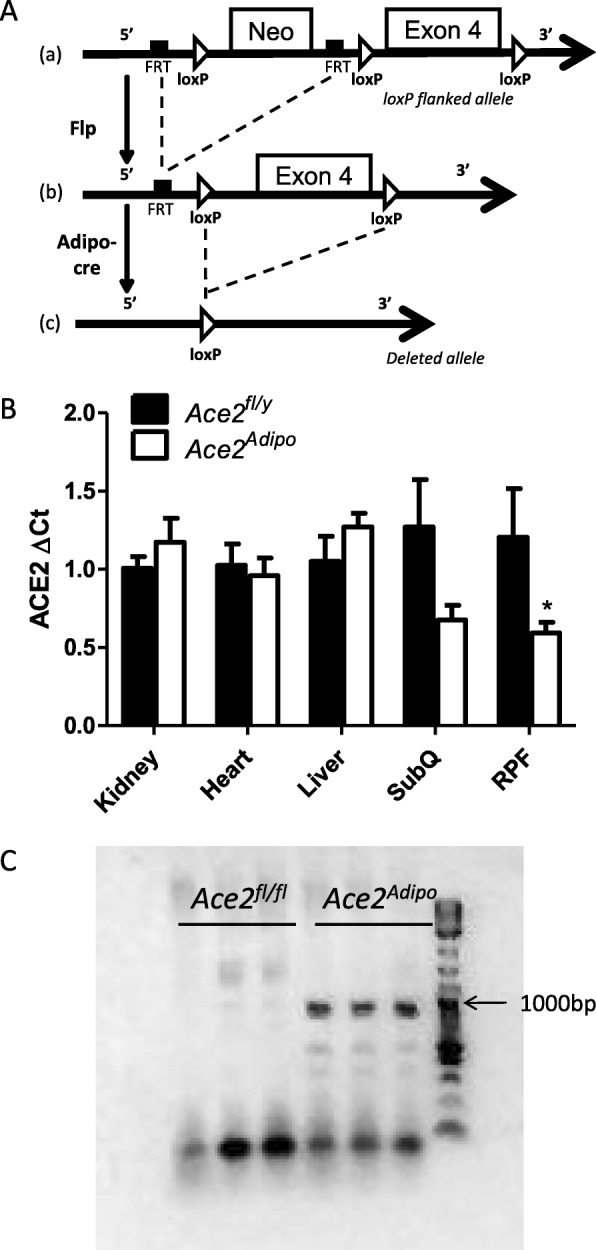
Development of a mouse model of adipocyte ACE2 deficiency. **a** Schematic representation depicting the loxP-flanked ACE2 allele before (**a**) and after successive recombination with Flp (**b**) and transgenic adiponectin-driven Cre expression (**c**). The disrupted allele is shown in **c**, indicating deletion of exon 4 of the ACE2 gene. **b** Tissue characterization demonstrating reduced ACE2 mRNA abundance is specific to adipose tissues (subcutaneous, SubQ; retroperitoneal, RPF) (*n* = 4–8 male mice/genotype). Data are mean + SEM; *P* < 0.05 compared to *Ace2*^*fl/y*^ using *t* test. **c** PCR reactions were performed with DNA extracted from RPF (*n* = 3 female mice/genotype). Primers amplify a 923 base pair product for the disrupted portion of the ACE2 gene

The ACE2 gene was deleted from adipose tissue using the Cre-Lox system driven by the adipocyte-specific promoter, adiponectin (Fig. 1a). ACE2 mRNA abundance was decreased by 47% in subcutaneous (SubQ) adipose tissue (*p* = 0.121), and by 51% in retroperitoneal fat (RPF, *p* < 0.05) from *Ace2*^Adipo^ compared to *Ace2*^*fl/y*^ mice (Fig. 1b). In contrast, there was no difference in ACE2 mRNA abundance in the kidney, heart, or liver from *Ace2*^*fl/y*^ compared to *Ace2*^Adipo^ mice (Fig. 1b). Deletion of ACE2 in *Ace2*^Adipo^ but not *Ace2*^*fl/fl*^ mice was confirmed by PCR in DNA extracted from RPF (Fig. 1c). Positive β-galactosidase staining was present in adipose tissues (epididymal [EF], RPF, and SubQ) of *Ace2*^Adipo^, but not *Ace2*^*fl/y*^ mice (Additional file 1: Figure S1). In contrast, no β-galactosidase staining was present in the liver, heart, or kidney of *Ace2*^*fl/fl*^ or *Ace2*^Adipo^ mice (Fig. 1d).

### Deficiency of ACE2 had no effect on the development of obesity in male or female mice

**Fig. 2:**
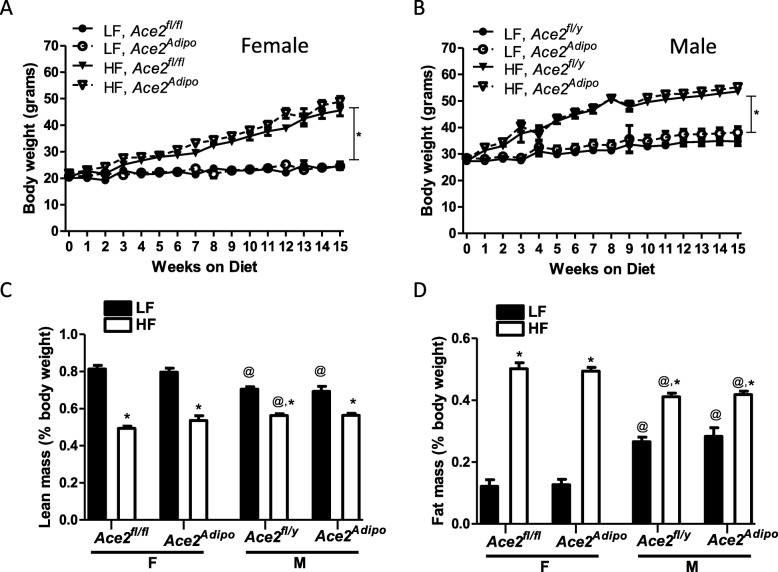
Deficiency of ACE2 in adipocytes has no effect on the development of obesity in male or female mice. Body weights (weekly) of female *Ace2*^*fl/fl*^ (**a**) or male *Ace2*^*fl/y*^ (**b**) and *Ace2*^Adipo^ mice fed a low fat (LF) or high fat (HF) diet. Lean mass (**c**) and fat mass (**d**) (as % body weight) of female or male mice from each genotype fed the LF or HF diet. Data are mean + SEM from *n* = 6–13 mice/genotype/diet. *p < 0.05 compared to LF within sex using two-way ANOVA followed by Holm-Sidak pairwise analysis; @*p* < 0.01 compared to female within diet group using two-way ANOVA followed by Holm-Sidak pairwise analysis

Both LF- and HF-fed male mice (Fig. 2b) had significantly greater body weights than female mice (Fig. 2a) throughout the study, independent of ACE2 genotype. After 15 weeks of diet feeding, body weight was increased significantly in HF-fed compared to LF-fed female and male mice (*p* < 0.001), with no differences in body weight between genotypes (Fig. 2a, b). In LF-fed mice of both genotypes, male mice had greater fat mass and less lean mass (as a percentage of body weight) compared to female mice (Fig. 2c, d; *p* < 0.001). In HF-fed mice of both genotypes, female mice had greater fat mass (as a percentage of body weight) compared to male mice (Fig. 2d; *p* < 0.001). While HF-feeding increased fat mass in both female and male mice (*p* < 0.01), the percent increase in fat mass was markedly higher in females (313% and 260% increase in *Ace2*^*fl/fl*^ and *Ace2*^Adipo^, respectively) compared to males (55% and 47% increase in *Ace2*^*fl/y*^ and *Ace2*^Adipo^, respectively), with no differences between genotypes. Lean mass percentage of body weight decreased in both female and male mice with HF-feeding (Fig. 2c; *p* < 0.01). Within genotypes, lean mass was greater in HF-fed male *Ace2*^*fl/y*^ (*p* < 0.01) but not *Ace2*^Adipo^ male mice compared to female counterparts (Fig. 2c).

### Ace2 deficiency in adipocytes increases SBP of HF-female mice to the level of wild-type HF-fed male mice

**Fig. 3:**
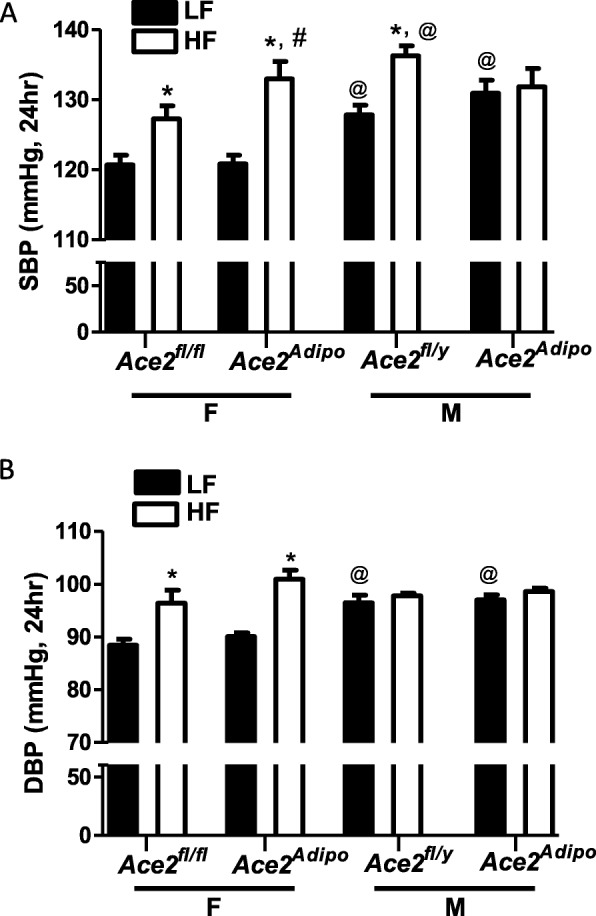
ACE2 deficiency in adipocytes increases blood pressures of obese female, but not obese male mice. Systolic blood pressures (SBP, 24-h average) (**a**) of female *Ace2*^*fl/fl*^ and male *Ace2*^*fl/y*^ and *Ace2*^Adipo^ mice fed a LF or HF diet for 4 months. Diastolic blood pressures (DBP) (**b**) of female and male mice of each genotype fed the LF or HF diet for 4 months. Data are mean + SEM from 4–5 mice/genotype/diet. *p < 0.01 compared to LF within sex using two-way ANOVA followed by Holm-Sidak pairwise analysis; #*p* < 0.05 compared to *Ace2*^*fl/fl*^ within sex group using two-way ANOVA followed by Holm-Sidak pairwise analysis; @*p* < 0.01 compared to female within diet group using two-way ANOVA followed by Holm-Sidak pairwise analysis

Male *Ace2*^*fl/y*^ mice had elevated SBP compared to female *Ace2*^*fl/fl*^ controls under both LF- and HF-fed conditions (24 h; Fig. 3a; *p* < 0.01). Similarly, DBP of LF-fed male *Ace2*^*fl/y*^ mice was also higher than LF-fed *Ace2*^*fl/fl*^ females (24 h; Fig. 3b; *p* < 0.001). In response to a HF diet, female *Ace2*^*fl/fl*^ mice had increased SBP and DBP compared to LF-fed *Ace2*^*fl/fl*^ female mice (Fig. 3a, b; *p* < 0.001). Male HF-fed *Ace2*^*fl/y*^ mice exhibited an increase in SBP, but not DBP, compared to male LF-fed *Ace2*^*fl/y*^ controls (Fig. 3a, b; *p* < 0.01 ).

Under LF feeding, male *Ace2*^Adipo^ mice had elevated SBP and DBP compared to LF-fed female *Ace2*^Adipo^ mice (Fig. 3a, b; *p* < 0.001). In response to the HF diet, female *Ace2*^Adipo^ mice exhibited an increase in SBP and DBP compared to LF-fed *Ace2*^Adipo^ females (Fig. 3a, b; *p* < 0.01). Moreover, SBP of HF-fed female *Ace2*^Adipo^ mice were elevated significantly compared to HF-fed *Ace2*^*fl/fl*^ females (Fig. 3a; *p* < 0.05). In contrast, there was no effect of HF diet on SBP or DBP in *Ace2*^Adipo^ male mice (Fig. 3a, b; *p* > 0.05). Moreover, deficiency of ACE2 in adipocytes of HF-fed females resulted in blood pressure levels (SBP and DBP) that were similar to those of HF-fed *Ace2*^*fl/y*^ male mice.

Female LF-fed mice, regardless of genotype, had significantly more physical activity than LF-fed males (Tables 1 and 2; 24 h, *p* < 0.01). HF-feeding resulted in a significant reduction in physical activity in females of each genotype (Table 1; *p* < 0.05). In contrast, there was no significant effect of HF-feeding on physical activity of male mice of either genotype (Table 2; *p* > 0.05). Heart rates of female mice were higher than males regardless of diet or genotype (Tables 1 and 2; *p* < 0.05). Moreover, HF feeding resulted in a significant increase in heart rate for each sex and genotype (Tables 1 and 2; *p* < 0.05).

**Table 1:** Telemetry parameters of female mice

	*Ace2* ^*fl/fl*^		*Ace2* ^Adipo^	
	LF	HF	LF	HF
24 h				
MAP (mmHg)	105.3 ± 1.1	112.4 ± 2.1^*^	106.1 ± 0.7	117.7 ± 2.1^, #^
Heart rate (bpm)	584 ± 8	617 ± 9^*^	563 ± 4^#^	622 ± 7^*^
Activity (counts/min)	12.8 ± 1.4	6.5 ± 0.6^*^	9.9 ± 1.5	6.4 ± 0.8^*^
PP (mmHg)	32.2 ± 1.2	30.8 ± 1.4	30.7 ± 1.5	32.0 ± 1.5
Light				
SBP (mmHg)	115.3 ± 1.1	122.3 ± 1.9^*^	114.9 ± 1.3	127.8 ± 2.5^*^
DBP (mmHg)	84.5 ± 0.8	92.7 ± 2.4^*^	85.2 ± 1.3	97.1 ± 1.0^*^
MAP (mmHg)	100.7 ± 0.7	100.8 ± 2.0^*^	100.8 ± 1.2	113.3 ± 2.1^*, #^
Heart rate (bpm)	576.6 ± 8.2	606.8 ± 9.3^*^	549.0 ± 4.5	610.8 ± 9.5^*^
Activity (counts/min)	7.1 ± 1.0	4.9 ± 0.4^*^	5.8 ± 0.7	4.3 ± 0.7
PP (mmHg)	30.8 ± 1.2	29.6 ± 1.3	29.7 ± 1.3	30.6 ± 1.3
Dark				
SBP (mmHg)	128.3 ± 0.4	134.3 ± 1.9	129.0 ± 1.7	139.7 ± 2.4^*^
DBP (mmHg)	94.0 ± 1.6	101.4 ± 2.8^*^	96.7 ± 0.6	105.3 ± 1.7^*^
MAP (mmHg)	111.8 ± 1.6	118.5 ± 2.2^*^	113.5 ± 0.9	123.6 ± 2.0^*^
Heart rate (bpm)	596.4 ± 8.1	634.0 ± 8.4^*^	578.9 ± 6.8	635.0 ± 7.0^*^
Activity (counts/min)	20.0 ± 1.8	8.9 ± 0.8^*^	15.1 ± 2.5	9.4 ± 1.2^*^
PP (mmHg)	34.4 ± 1.3	32.9 ± 1.5	32.2 ± 1.7	33.8 ± 1.4

*PP* pulse pressure, *SBP* systolic blood pressure, *DBP* diastolic blood pressure, *MAP* mean arterial pressure

Data are mean ± SEM (*n* = 4–5 mice/diet/genotype)

*p < 0.05 compared to LF within genotype following pairwise statistical analysis

#*p* < 0.05 compared to *Ace2*^*fl/fl*^ within the diet group following pairwise statistical analysis

**Table 2:** Telemetry parameters of male mice

	*Ace2* ^*fl/y*^		*Ace2* ^Adipo^	
	LF	HF	LF	HF
24 h				
MAP (mmHg)	112.5 ± 0.8	117.7 ± 0.7^*^	114.1 ± 0.9	115.3 ± 1.2
Heart rate (bpm)	556.7 ± 6.7	582.2 ± 7.5^*^	559.7 ± 6.5	581.1 ± 14.2^*^
Activity (counts/min)	5.9 ± 0.7	5.0 ± 0.5	5.6 ± 0.8	5.5 ± 0.7
PP (mmHg)	31.3 ± 2.1	38.2 ± 1.0	33.8 ± 2.0	33.18 ± 2.8
Light				
SBP (mmHg)	120.9 ± 1.3	130.0 ± 1.6^*^	123.88 ± 1.6	128.8 ± 2.5
DBP (mmHg)	91.6 ± 0.6	93.3 ± 1.1	91.9 ± 1.2	94.6 ± 1.6
MAP (mmHg)	106.3 ± 0.4	112.2 ± 1.2^*^	108.1 ± 0.8	110.9 ± 1.5
Heart rate (bpm)	524.3 ± 5.2	555.3 ± 10.1^*^	534.6 ± 11.1	561.4 ± 13.0
Activity (counts/min)	3.0 ± 0.3	3.3 ± 0.5	3.0 ± 0.4	3.5 ± 0.2
PP (mmHg)	29.2 ± 1.9	35.6 ± 0.8	31.9 ± 1.9	32.3 ± 2.5
Dark				
SBP (mmHg)	139.0 ± 1.3	142.1 ± 2.3	140.5 ± 2.5	134.5 ± 3.7
DBP (mmHg)	105.3 ± 2.1	104.3 ± 1.2	104.1 ± 1.0	102.5 ± 1.6
MAP (mmHg)	122.3 ± 1.3	125.4 ± 1.7	122.4 ± 1.4	120.4 ± 2.2
Heart rate (bpm)	598.6 ± 10.6	621.3 ± 7.7	595.3 ± 3.7	614.9 ± 15.0
Activity (counts/min)	10.8 ± 1.1	7.3 ± 0.7	9.2 ± 1.4	7.4 ± 1.7
PP (mmHg)	33.7 ± 2.5	40.9 ± 1.4	36.4 ± 2.2	35.1 ± 3.0

*PP* pulse pressure, *SBP* systolic blood pressure, *DBP* diastolic blood pressure, *MAP* mean arterial pressure

Data are mean ± SEM (*n* = 4–5 mice/diet/genotype)

*p < 0.05 compared to LF within genotype following pairwise statistical analysis

#*p* < 0.05 compared to *Ace2*^*fl/y*^ within the diet group following pairwise statistical analysis

### SBP response to acute AngII challenge is augmented in obese female mice with adipocyte-ACE2 deficiency

**Fig. 4:**
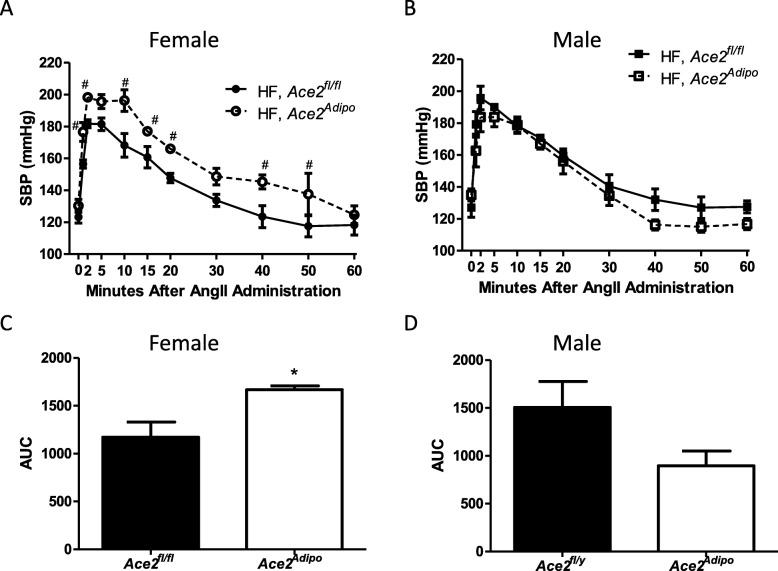
Systolic blood pressure (SBP) response to acute AngII challenge is augmented in obese female, but not obese male mice with adipocyte-ACE2 deficiency. At 4 months of HF feeding, the time course of SBP following an acute injection (sc) of AngII (20 μg/kg) to female *Ace2*^*fl/fl*^ (**a**) or male *Ace2*^*fl/y*^ (**b**) and *Ace2*^Adipo^ mice. Data are reported as the average blood pressure per minute at each time point. Integrated area under the curve (AUC) corresponding to the time course of SBP response to AngII for HF-fed female *Ace2*^*fl/fl*^ (**c**) or HF-fed male *Ace2*^*fl/y*^ (**d**) and *Ace2*^Adipo^ mice. Data are mean + SEM for *n* = 4 mice/genotype. #*p* < 0.05 compared to *Ace2*^*fl/fl*^ at each time point using repeated measures (RM) two-way ANOVA; *p < 0.05 compared to *Ace2*^*fl/fl*^ using *t* test

Previous studies demonstrated that adipocyte-derived AngII contributes to increased SBP of HF-fed male mice [7]. AngII is a substrate for ACE2. Therefore, we challenged HF-fed *Ace2*^*fl/fl*^ and *Ace2*^Adipo^ male and female mice with a single dose of the ACE2 substrate, AngII (20 μg/kg body weight, subcutaneous) and quantified blood pressure. In male and female mice of each genotype, SBP was increased by administration of AngII, with a rapid peak blood pressure effect within 2 minutes of AngII administration; Fig. 4a, b). Female HF-fed *Ace2*^Adipo^ mice exhibited an increased maximal blood pressure response to AngII (Fig. 4a; *p* < 0.05) that was extended in duration compared to HF-fed *Ace2*^*fl/fl*^ females, as evidenced by an increased AUC (blood pressure response above baseline through 60 min; Fig. 4c; *p* < 0.05). In contrast, there was no significant effect of adipocyte ACE2 deficiency on the maximal response or duration of the blood pressure response to AngII between HF-fed male *Ace2*^*fl/y*^ and *Ace2*^Adipo^ mice (Fig. 4b, d).

### In obese transwomen administered 17β-estradiol, increased plasma Ang-(1-7)/AngII balance is inversely correlated with changes in SBP

**Fig. 5:**
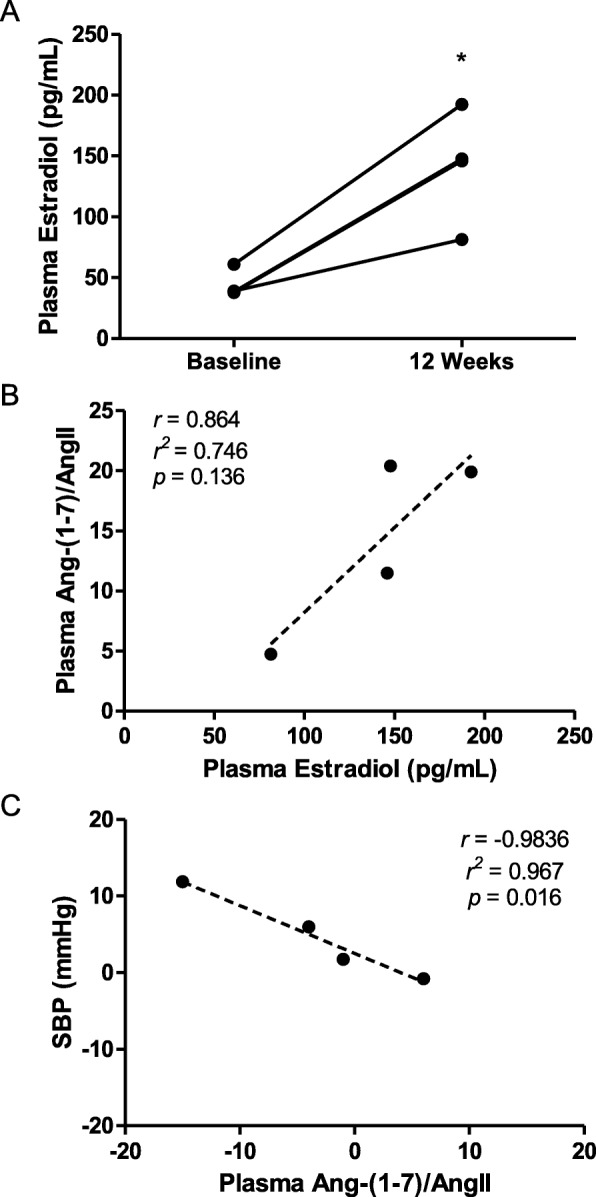
Administration of 17β-estradiol to obese transwomen initiating gender-affirming hormone therapy increases plasma concentrations of 17β-estradiol, which correlates positively to plasma Ang-(1-7)/AngII balance and negatively to systolic blood pressures (SBP). **a** Plasma 17β-estradiol concentrations before (baseline) and after administration of 17β-estradiol to obese transwomen for 12 weeks. **b** Scatterplot showing the correlation between the ratio of Ang-(1-7) to AngII concentrations in plasma to plasma 17β-estradiol concentrations after 12 weeks of 17β-estradiol administration. **c** Scatterplot showing correlation between change in SBP and the ratio of Ang-(1-7) to AngII in plasma following 12 weeks 17β-estradiol administration. *N* = 4 subjects. *p < 0.05 compared to baseline

We sought to translate findings from experimental mice to humans and therefore examined effects of 12 weeks of estradiol therapy on plasma Ang-(1-7)/AngII balance and SBP in obese transwomen initiating gender-affirming hormone therapy (*n* = 4 subjects). Body mass index (BMI), as an index of obesity, was not significantly influenced by estradiol administration (Table 3; *p* > 0.05). As anticipated, plasma concentrations of estradiol were increased significantly with estradiol treatment compared to baseline estradiol concentrations (Fig. 5a; *p* < 0.05), although estradiol levels in one subject did not reach the target estradiol levels for gender-affirming hormone therapy (81.3 pg/mL vs target range of 90–200 pg/mL). Plasma concentrations of individual components of the RAS (angiotensinogen, renin, AngII, Ang-(1-7)) were not significantly influenced by estradiol administration compared to baseline values (Table 3; *p* > 0.05). The ratio of plasma concentrations of Ang-(1-7) to AngII, a surrogate for ACE2 activity, was increased 2.57-fold with estradiol compared to baseline, but this effect was not statistically significant (Table 3; *p* = 0.19). Moreover, after 12 weeks of estradiol administration, the balance of Ang-(1-7)/AngII in plasma correlated positively to plasma estradiol concentrations, although the correlation was not statistically significant (Fig. 5b; *r*^2^ = 0.746; *p* = 0.136). In addition, after 12 weeks of estradiol administration, the increase in plasma Ang-(1-7)/AngII balance correlated significantly to reductions of SBP (Fig. 5c; *r*^2^ = 0.967; *p* = 0.016).

**Table 3:** Characteristics of obese, transgendered women at baseline, and 12 weeks following oral estradiol therapy.

Parameter	Baseline	12 weeks of estradiol
BMI (m/kg^2^)	37.5 ± 3.4	38.1 ± 3.4
Plasma renin activity (ng/mL*h)	0.85 ± 0.25	0.76 ± 0.25
Plasma angiotensinogen (μg/mL)	17.0 ± 3.9	20.8 ± 5.0
Plasma AngII (pg/mL)	42.3 ± 9.0	40.8 ± 14.6
Plasma Ang-(1-7) (ng/mL)	0.36 ± 0.05	0.42 ± 0.02
Ratio of Ang-(1-7)/AngII (pg/mL)	5.53 ± 1.74	14.13 ± 3.73

## Discussion

This study investigated the role of adipocyte ACE2 in the differential regulation of blood pressure in female versus male mice with diet-induced obesity. We translated these experimental findings to humans by studying the effects of estradiol therapy in transwomen on the plasma Ang-(1-7)/AngII balance and blood pressure. The major findings of these studies are (1) deficiency of ACE2 in adipocytes increased SBP in obese female, but not male mice, (2) obese female, but not male mice with adipocyte ACE2 deficiency exhibit an augmented blood pressure response to the ACE2 substrate, AngII, (3) administration of estradiol as part of gender-affirming hormone therapy in a small, proof-of-principle study led to higher plasma Ang-(1-7)/AngII balance, which correlates inversely to change in systolic blood pressure in obese transwomen. These data demonstrate a role for adipocyte ACE2 in protection of female mice from obesity-hypertension. Moreover, since the human study demonstrated a correlation between serum estradiol concentrations and the systemic Ang-(1-7)/AngII balance and blood pressure, these results suggest that positive regulation of ACE2 by estrogen may serve as a potential protective mechanism against obesity-hypertension in females.

Obesity is a prominent risk factor for the development of hypertension. Despite the increased prevalence of obesity in women versus men, premenopausal women have a lower prevalence of hypertension, suggesting that the blood pressure-elevating effects of obesity are lower in women. Polymorphisms of ACE2 have been linked to essential hypertension in females [15]. A recent study demonstrated that systemic ACE2 activity levels were negatively correlated with BMI and blood pressure in female essential hypertension patients [16]. Previous studies from our laboratory demonstrated that plasma Ang-(1-7) concentrations were higher in obese normotensive female mice than obese hypertensive male mice and were associated with increased adipose tissue expression and activity of ACE2. In contrast, ACE2 expression and activity in the kidney, a site with considerable expression of the Ang-(1-7) peptide-forming enzyme, were not altered in obese male or obese female mice compared to lean controls. Whole-body deficiency of ACE2 increased blood pressure in obese male mice and converted female obese mice to a hypertensive phenotype [11]. Moreover, obese hypertensive ACE2 deficient females exhibited reductions in plasma concentrations of Ang-(1-7) [11]. However, the cell type responsible for effects of whole-body ACE2 deficiency to promote obesity-hypertension in both sexes was not identified. Findings from the present study extend previous results by demonstrating that adipocyte ACE2 contributes to protection from obesity-hypertension in females, but not in obese males. Since previous findings demonstrated that whole body deficiency of ACE2 increased SBP in both obese female and male mice [11], these results suggest that effects of ACE2 deficiency to promote obesity-hypertension in males were not adipocyte-mediated. In contrast, our results suggest that in obese females, adipocytes are the predominant source of ACE2 for the development of obesity-hypertension.

We demonstrated previously that 17β-estradiol promoted ACE2 mRNA expression in 3T3-L1 adipocytes by eliciting ERα-binding to the ACE2 promoter [12]. Further, administration of 17β-estradiol to obese ovariectomized female mice reduced blood pressure and stimulated ACE2 activity and mRNA abundance in adipose tissue, while having no effect on blood pressures of obese ACE2-null females [12]. In this study, deletion of ACE2 in adipocytes increased blood pressures of obese female mice, but had no effect on blood pressures of obese male mice. These findings are consistent with published reports of estrogen regulation of the ACE2/Ang-(1-7) axis, which would support a sex-specific effect of adipocyte ACE2. For example, in ovariectomized hypertensive rats, administration of 17β-estradiol reduced blood pressure and promoted the production of Ang-(1-7) [17]. In a renal wrap model of hypertension in female rats, 17β-estradiol administration to ovariectomized female rats with renal wrap hypertension upregulated renal ACE2 expression and activity and reduced renal injury [18]. However, it is possible that testosterone also regulates ACE2 expression, since renal ACE2 activity was demonstrated to be higher in male compared to that in female mice [11, 19]. Moreover, since previous findings demonstrated that ACE2 activity was increased by obesity in adipose tissue of female, but not male mice [11], then these results suggest that obesity per se may introduce sex- and tissue-specific regulation of ACE2. Regardless, results from the present study indicate a primary role for adipocyte ACE2 in the development of obesity-hypertension in females.

An interesting finding of the present study was an augmented response to an acute blood pressure challenge with AngII in female, but not in male obese mice with adipocyte ACE2 deficiency. Since AngII is a substrate of ACE2, then these results suggest that adipocyte ACE2 deficiency either influences the systemic half-life of AngII and the balance of Ang-(1-7)/AngII, or that local conversion of systemic AngII to Ang-(1-7) by adipocyte ACE2 regulates blood pressure. In agreement, previous findings from our laboratory demonstrated that adipocyte expression of angiotensinogen, the precursor to AngII, influences systemic concentrations of AngII and the development of obesity-hypertension in male mice [7]. These results suggest that local expression of components of the RAS can influence systemic levels of these components and the circulating production of angiotensin peptides. In support, the liver was demonstrated as a primary source of renal AngII [20], and hepatic deficiency of angiotensinogen was demonstrated to influence adipose explant production of angiotensinogen in obese male mice [7]. It is unclear from the present study if adipocyte ACE2 influences the systemic half-life of AngII; however, results from this study demonstrate that adipocyte ACE2 regulates the blood pressure response to an acute systemic AngII challenge.

In normotensive humans, blood pressure is higher in males than in females [21]. This finding is consistent with studies in rodents, where normotensive male rats have higher blood pressures than female rats [22]. Our results extend these and other findings [11] by demonstrating that obese female wild type mice have lower blood pressures than obese males. Blood pressure is thought to be directly related to adiposity [9]. Thus, it is noteworthy that HF-fed females had more adiposity than HF-fed males, but yet had lower blood pressures than obese males. These findings suggest that the more expanded fat mass of HF-fed females results in the potential production of adipocyte-derived factors, such as ACE2, that protect against obesity-hypertension. Alternatively, the presence of estrogens in obese females augments the production of protective factors, such as adipocyte ACE2, to blunt the development of obesity-hypertension. Additional vasoprotective effects of estrogen include induction of nitric oxide to promote vasodilation [23] and blunting of vasoconstrictor effects mediated by the sympathetic nervous system [24]. Even with obesity, estrogen may have positive metabolic effects such as increased energy expenditure, regulation of food intake, and inhibition of adipogenesis [25]. Thus, declining estrogen levels resulting in both increasing body weight and loss of vascular protection may contribute to the rise in hypertension post-menopause.

To translate these findings from mice to humans, we performed a proof-of-principle study in obese transwomen initiating gender-affirming hormone therapy with 17β-estradiol. Approximately 1.4 million persons in the USA, or 0.6% of adults, identify as transgender [26, 27]. Unfortunately, the cardiovascular health of transgender persons taking cross-sex hormone therapies long-term is largely unknown. In this study, we focused on obese transgender women before and after initiation of 17β-estradiol administration for 3 months as part of standard transgender therapy. Notably, participation in this study required delaying the use of spironolactone for 12 weeks, which limited participation. Previous studies found that in 21 transgender women taking 17β-estradiol (2–6 mg/day) for 5 years, plasma estradiol levels rose from 108 to 237 pmol/L, and systolic blood pressure decreased from 119 to 112 mmHg [28]. We recently demonstrated a negative correlation between BMI and estradiol dose needed to achieve target estradiol levels of 90–200 pg/mL [29], likely due to higher estradiol levels found in obese males from aromatization of androgens to estrogens in adipose tissue [30–32]. In agreement, results from this study demonstrate mean plasma17-β estradiol concentrations reached the target estradiol levels despite use of a fairly low dose of 17β-estradiol (1–2 mg/day) in obese transwomen. In the current study, we report 12 weeks of estradiol therapy had no significant effect to modulate individual components of the RAS, which is at odds with published literature reporting significant systemic alterations of the RAS by estradiol [33]. However, studies of estrogen’s influence on the RAS in humans are largely based on hormonal changes throughout the menstrual cycle [34], during pregnancy [35], or with estrogen-replacement therapy [36] in cisgender women. To our knowledge, these are the first studies examining effects of 17β-estradiol administration to obese transwomen on indices of the systemic RAS. Moreover, our results extend previous findings by demonstrating an association between 17β-estradiol levels, plasma Ang-(1-7)/AngII balance, and systolic blood pressures of transwomen.

There are several limitations to the clinical study. First, there were challenges in recruitment due to participation requiring a delay in use of spironolactone therapy for its anti-androgenic effects. Second, we do not have measures of testosterone levels in these transwomen; testosterone was not measured as the literature reports inconsistent effects of estradiol on testosterone levels [37, 38], and there is no evidence that testosterone levels affect desired body changes of gender-affirming hormone therapy. Third, these measures were drawn only once after only 12 weeks of therapy, and although mean plasma 17β-estradiol levels achieved the target of 90–200 pg/mL, not all subjects achieved a plasma 17β-estradiol level in the target range on their initial prescribed dose of estradiol. Further changes in the RAS may occur over more prolonged therapy. Finally, we were unable to determine if adipose ACE2 contributes to the observed associations between systemic 17β-estradiol concentrations, plasma Ang-(1-7)/AngII balance, and blood pressure. However, taken together, the murine and clinical data support estrogen regulation of ACE2 as a contributor to blood pressure regulation in the development of obesity-hypertension.

## Conclusions

In conclusion, these results demonstrate that deficiency of ACE2 in adipocytes augments the development of hypertension and the pressor response to AngII in obese female, but not obese male mice. These results suggest that adipocyte ACE2 protects female mice from the development of obesity-hypertension. Moreover, translation of these findings to obese transwomen demonstrates a negative association among plasma Ang-(1-7)/AngII balance and systolic blood pressure with increased plasma 17β-estradiol concentrations. Taken together, these results suggest that adipocyte-derived ACE2 regulates the balance of vasodilator (Ang-(1-7) to vasoconstrictor (AngII) angiotensin peptides and contributes to sex differences in obesity-hypertension.

## Additional file

Additional file 1: **Figure S1.** Positive β-galactosidase staining in adipose tissue of mice with adipocyte deficiency of ACE2. (PPTX 922 kb)

## Data Availability

The datasets used and/or analyzed during the current study are available from the corresponding author on reasonable request.
