# Supplementary material for: Adipocyte deficiency of ACE2 increases systolic blood pressures of obese female C57BL/6 mice
Source: Biol Sex Differ. 2019 Sep 4;10:45. doi: 10.1186/s13293-019-0260-8 (PMC6727421; doi:10.1186/s13293-019-0260-8)
Supplement: Supplementary file 1 — Figure S1. Positive β-galactosidase staining in adipose tissue of mice with adipocyte deficiency of ACE2. (PPTX 922 kb) [file 13293_2019_260_MOESM1_ESM.pptx]

## Slide 1
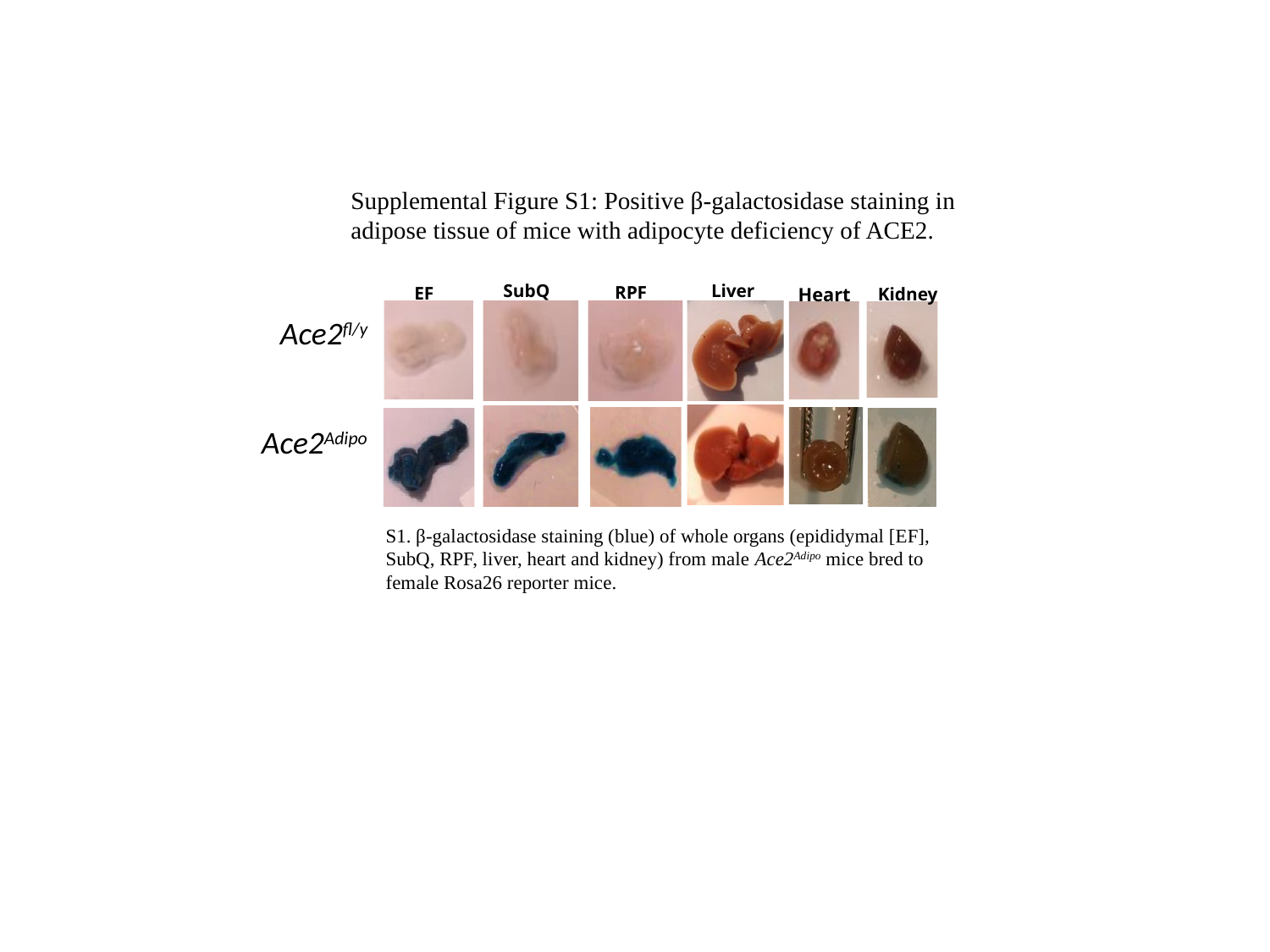

Supplemental Figure S1: Positive β-galactosidase staining in adipose tissue of mice with adipocyte deficiency of ACE2.
Liver
SubQ
RPF
EF
Kidney
Heart
Ace2fl/y
Ace2Adipo
S1. β-galactosidase staining (blue) of whole organs (epididymal [EF], SubQ, RPF, liver, heart and kidney) from male Ace2Adipo mice bred to female Rosa26 reporter mice.
